# Aberrant functioning of the theory-of-mind network in children and adolescents with autism

**DOI:** 10.1186/s13229-015-0052-x

**Published:** 2015-10-27

**Authors:** Rajesh K. Kana, Jose O. Maximo, Diane L. Williams, Timothy A. Keller, Sarah E. Schipul, Vladimir L. Cherkassky, Nancy J. Minshew, Marcel Adam Just

**Affiliations:** Department of Psychology, University of Alabama at Birmingham, CIRC 235G, 1719 6th Ave South, Birmingham, AL 35294-0021 USA; Department of Speech-Language Pathology, Duquesne University, Pittsburgh, PA USA; Department of Psychology, Carnegie Mellon University, Pittsburgh, PA USA; Department of Psychiatry, University of Pittsburgh, Pittsburgh, PA USA

**Keywords:** Theory-of-mind, Autism, fMRI, Functional connectivity MRI, mentalizing

## Abstract

**Background:**

Theory-of-mind (ToM), the ability to infer people’s thoughts and feelings, is a pivotal skill in effective social interactions. Individuals with autism spectrum disorders (ASD) have been found to have altered ToM skills, which significantly impacts the quality of their social interactions. Neuroimaging studies have reported altered activation of the ToM cortical network, especially in adults with autism, yet little is known about the brain responses underlying ToM in younger individuals with ASD. This functional magnetic resonance imaging (fMRI) study investigated the neural mechanisms underlying ToM in high-functioning children and adolescents with ASD and matched typically developing (TD) peers.

**Methods:**

fMRI data were acquired from 13 participants with ASD and 13 TD control participants while they watched animations involving two “interacting” geometrical shapes.

**Results:**

Participants with ASD showed significantly reduced activation, relative to TD controls, in regions considered part of the ToM network, the mirror network, and the cerebellum. Functional connectivity analyses revealed underconnectivity between frontal and posterior regions during task performance in the ASD participants.

**Conclusions:**

Overall, the findings of this study reveal disruptions in the brain circuitry underlying ToM in ASD at multiple levels, including decreased activation and decreased functional connectivity.

**Electronic supplementary material:**

The online version of this article (doi:10.1186/s13229-015-0052-x) contains supplementary material, which is available to authorized users.

## Background

Theory-of-mind (ToM) underlies the ability to explain and predict the behavior of others by attributing to them specific mental states such as their intentions, beliefs, desires, or emotions [1]. ToM also influences the quality of social interactions by enabling people to effectively navigate the interpersonal world and make common sense explanations of others’ behavior. Functional magnetic resonance imaging (fMRI) studies in typically developing (TD) individuals have identified a frontal-posterior network that activates during ToM tasks, which includes the medial prefrontal cortex (MPFC), the posterior cingulate cortex (PCC), and bilateral temporoparietal junction (LTPJ and RTPJ) [2, 3]. It should be noted that while these regions have been central to the ToM network, regions such as the right anterior superior temporal sulcus and medial precuneus have also been implicated in processing ToM [4, 5]. Each of these core regions (MPFC, PCC, LTPJ, RTPJ) has been implicated in several specific social cognitive processes. For instance, the MPFC has been associated with reflective reasoning about actions and judgments, including goals and intentions [6, 7], the PCC with visual imagery, retrieval of episodic information, and self-projection [8, 9], and bilateral TPJ with making transient mental inferences (goals, beliefs) about people [10].

Impairment in social interactions is one of the defining characteristics of autism spectrum disorders (ASD) [11, 12], which may underlie a deficit in social cognition in general, and a deficit in ToM in particular [13–17]. Deficits in ToM can have detrimental effects on social cognition and can carry its effects into other domains of cognitive functioning in individuals with ASD [18]. Functional MRI studies have complemented the behavioral findings of poor ToM skills by reporting altered brain activity during ToM tasks in ASD [4, 10, 19–27]. More recently, functional connectivity MRI (fcMRI), which assesses the correlation of the fMRI blood-oxygen-level dependent (BOLD) time-series of a pair of brain areas, has been used by some of these studies to characterize the neural circuitry underlying ToM in ASD [22–25, 27]. Overall, there is an emerging consensus that aberrant activity and connectivity among different brain regions during ToM and other social-cognitive functions can be considered a strong neurobiological feature of brain functioning in ASD [18, 28–32].

Many previous fMRI studies examining connectivity of the ToM network have found reduced functional connectivity between frontal and more posterior regions in adults with ASD, which may entail disrupted functional integration among core regions underlying such tasks [28]. Since ToM is a complex function mediated by the topographically distributed ToM network including four key nodes (PCC, MPFC, LTPJ, RTPJ), coordination within this network and across other centers may be critical in accomplishing social tasks. While weaker synchronization among these regions has been previously found in individuals with ASD during ToM tasks [10, 21, 23, 24], reduced connectivity may not be a universal feature of autism due to its heterogeneity [33, 34]. In addition, children with ASD may show a different connectivity profile than adults with ASD [35, 36], though this still remains a topic of debate [29]. It should be noted that methodological differences across neuroimaging studies have affected the consistency, reliability, and replicability of functional connectivity findings in ASD [37]. While functional connectivity patterns in adults with ASD may provide a snapshot of how the disorder has progressed and affected the brain over several years, it is equally important to understand the nature of connectivity during childhood and adolescence for a better characterization of the developmental profile of this disorder.

The current study examines ToM network connectivity in children and adolescents with ASD using animations involving the complex movement of geometrical shapes in ways that often evoke a social interpretation of their interaction. These animations have been used successfully in a growing number of studies to investigate ToM and attribution of agency in individuals with and without ASD (see [38] for an extensive review). In autism, such stimuli have been used in two previous neuroimaging studies of ToM in adults, both of which found underconnectivity in participants with autism [21, 23]. The purpose of the current study is to assess the brain responses involved in processing ToM in children and pre-adolescents with ASD using region of interest (ROI) analysis as well as whole-brain analysis. Thus, activation and functional connectivity patterns across different levels of analyses are examined. ROI analyses involved regions from the ToM network, as well as additional regions such as the superior temporal sulcus (STS) due to its role in social perception [39], regions considered part of the substrate for action understanding, such as the inferior frontal gyrus [6], angular gyrus [40], and cerebellum, a region that has increasingly shown, of late, to be involved in social cognition [41]. We hypothesize atypical brain activation and functional connectivity in children and adolescents with ASD. This study is novel in that it uses dynamic non-verbal stimuli to examine the integrity of the ToM network in pre-adult ASD, a relatively under-studied topic. The findings of this study will provide valuable insight into understanding the functional makeup of an important neural network that is critical in characterizing the social symptoms in individuals with ASD.

## Methods

### Participants

Thirteen high-functioning children and adolescents with autism (mean age *=* 12.6 years, range = 10–16 years) and thirteen TD control individuals (mean age *=* 12.7 years, range = 10–15 years) were included in this study (all with full-scale and verbal IQ scores of 75 or above). Participants were matched as a group on the basis of age, gender, handedness, and IQ (Table 1). IQ was assessed in all participants using the Wechsler Abbreviated Scale of Intelligence (WASI-I) [42]. The diagnosis of autism, as per DSM-IV guidelines [43], was established using two structured research diagnostic instruments, the Autism Diagnostic Interview-Revised (ADI-R) [44] and the Autism Diagnostic Observation Schedule-Generic (ADOS-G) [45], supplemented with confirmation by expert opinion. Potential participants with autism were excluded on the basis of an associated infectious or genetic disorder, such as fragile-X syndrome or tuberous sclerosis. Potential control participants and participants with autism were also excluded if found to have evidence of birth asphyxia, head injury, or a seizure disorder. Exclusionary criteria were based on neurologic history, examination, and chromosomal analysis. Potential control participants were also screened to exclude those with medical illnesses or a family history of autism, developmental cognitive disorder, affective disorder, anxiety disorder, schizophrenia, obsessive compulsive disorder, or other neurologic or psychiatric disorders thought to have a genetic component (in first-degree relatives or self). They were also excluded based on medications that affect the central nervous system, hypertension, diabetes, substance abuse (self or first-degree relative), steroid use (extreme use such as steroids used in inhalers for asthmatics), and autism in first-, second-, or third-degree relatives. Each participant’s parents signed an informed consent, and written assent was obtained from all minor participants. These documents and procedures had been approved by the University of Pittsburgh and Carnegie Mellon University Institutional Review Boards (Table 1).

**Table 1:** Demographic information

	Groups		*p* value
	TD (*n* = 13)	ASD (*n* = 13)	
Gender	11 M, 2 F	11 M, 2 F	–
Handedness	12 R, 1 L	12 R, 1 L	–
Age (in years)	12.7 (±1.5; 10–15)	12.6 (±1.9; 10–16)	0.83
Verbal IQ	103.7 (±10.1; 87–120)	98.2 (±13.8; 82–119)	0.26
Performance IQ	107.8 (±7.6; 94–120)	103.2 (±17.8; 89–132)	0.40
Full-scale IQ	106.9 (±8.7; 93–119)	100.8 (±15.2; 83–128)	0.25
ADOS-G			
- Communication	–	4.0 (±0.9; 3–5)	–
- Social	–	9.3 (±1.6; 6–11)	–
- Socio-communicative	–	13.3 (±1.7; 3–16)	–
ADI-R (in months)			
- Word	–	24.3 (±10.0; 10–48)	–
- Phrase	–	113.3 (±278; 12–997)	–

Values are presented as mean (standard deviation; range). The *p* value is independent *t* tests for differences between groups. ADOS scores were not available for one individual

*TD* typically developing, *ASD* autism spectrum disorder, *M* male, *F* female, *R* right, *L* left

### Experimental paradigm

This experiment compared the brain activation of autism and TD control participants while they were interpreting the behaviors of two interacting animated figures (a large red triangle and a small blue triangle) commonly known as the Frith-Happé animations [38, 46]. For example, if the red triangle’s movement mirrored the blue triangle’s movement but with some delay, the red triangle could be interpreted to be *chasing* the blue triangle. Three types of animations were used: theory-of-mind (ToM), goal-directed (GD), and random (RD). In the ToM condition, the geometrical figures moved in a way that could be interpreted as driven by an intentional action or interaction involving thoughts and feelings (e.g., coaxing). In the GD condition, the geometrical figures engaged in an interaction with each other in a simple purposeful level (e.g., chasing). In the RD condition, the geometrical figures did not engage with each other at all (e.g., two individual tennis ball movements). There were three epochs each of ToM, GD, and RD stimuli, each containing one animation. The basic visual characteristics depicted in the three types of animations were similar in terms of shape, overall speed of motion, orientation changes, and total duration (between 26 and 47 s).

After the presentation of each animation, four single-word response alternatives that described different actions were presented on the screen and participants were asked to make a forced-choice judgment about which of the words best described each animated action. The correct response was always an accurate description of the animation (determined as the most frequently generated description in a norming study). Another response alternative was an inaccurate description of the animation, but of the appropriate category (e.g., in the ToM condition, this response referred to a mental state, but an incorrect mental state). The other two answers were inaccurate descriptions of the animation that could have applied to animations in the other two conditions (i.e., in the ToM condition, these incorrect response alternatives referred to a random motion and a goal-directed motion; in the GD condition, they referred to a ToM motion and a random motion; and in the RD condition, they referred to a ToM motion and a goal-directed motion). For example, for a ToM animation that depicted “coaxing,” the foils were pushing (GD), surprising (ToM), and spinning (RD). Participants made their responses using two two-button mice, one held in each hand. Each button corresponded to one of the four multiple-choice answers. Responses were accepted for 15 s from the end of each animation. The presentation of each animation constituted a separate event in the experimental design. The animations were presented in blocks of three, one from each condition, with a separation of 6 s between trials within a block. The onset of each animation was synchronized with the beginning of a TR. A 30-s fixation interval was presented between each block, with participants instructed to stare at a centered asterisk and relax.

### fMRI acquisition

All imaging data were acquired at the Brain Imaging Research Center (BIRC), jointly administered by Carnegie Mellon University and the University of Pittsburgh on a 3T Siemens Allegra scanner. The stimuli were rear-projected onto a semi-translucent plastic screen, and participants viewed the screen through a mirror attached to the head coil. For the functional imaging, a gradient echo, echo-planar pulse sequence was used with TR = 1000 ms, TE = 30 ms, and a flip angle of 60°. Sixteen adjacent oblique-axial slices were acquired in a single cycle of scanning in an interleaved sequence, with a 5-mm slice thickness, a 1-mm slice gap, a 20 × 20 cm FOV, and a 64 × 64 matrix, resulting in an in-plane resolution of 3.125 × 3.125 mm, for a total of 666 volumes (11 min and 6 s). A 160-slice 3D MPRAGE volume scan with TR = 200 ms, TE = 3.34 ms, flip angle = 7°, FOV = 25.6 cm, 256 × 256 matrix size, and 1-mm slice thickness was acquired at the same orientation as the oblique-axial functional images.

### Data preprocessing

Functional images were processed using a combination of Analysis of Functional NeuroImages (AFNI) software [47] and the Oxford Centre for Functional Magnetic Resonance Imaging of the Brain (FMRIB) Software Library (FSL) [48]. Functional images were slice-time corrected, and correction for head motion was performed by registering each functional volume to the first time point of the scan using AFNI’s *3dvolreg*. These images were then registered to the anatomical images via FSL’s linear image registration tool *FLIRT* [49, 50]. Both images were resampled (3 mm isotropic) and standardized to Montreal Neurological Institute (MNI) space via FSL’s nonlinear registration tool (*FNIRT*), and a Gaussian spatial smoothing filter with a global full width at half maximum (FWHM) of 8 mm was applied using AFNI’s *3dBlurToFWHM*.

### fMRI activation analysis

Functional images were individually scaled to a mean of 100, and statistical analysis was performed on individual data by using a general linear model (GLM) via AFNI’s *3dDeconvolve* with ToM, GD, RD, ToM question (ToMQ), goal-directed question (GDQ), and random question (RDQ) trials as regressors of interest. Each condition was modeled using a duration-modulated (between 26 and 47 s) hemodynamic response function (the dmBLOCK option in *3dDeconvolve*). Six additional rigid-body motion parameters acquired from motion estimation were modeled as nuisance covariates in the GLM. The following orthogonal contrasts were computed to assess average differences in brain response, based on the interest of our study: theory-of-mind vs. random animation (ToM + ToMQ vs. RD + RDQ) and goal-directed vs. random Animation (GD + GDQ vs. RD + RDQ)*.*

ROI analysis was first conducted using regions considered part of the ToM network (MPFC, PCC, and bilateral TPJ) along with other regions associated with social perception (bilateral STS and cerebellum) and action understanding (bilateral inferior frontal and angular gyrus). The ROIs for this analysis were obtained using an independent whole-brain activation map (for the contrast social ToM > RD) derived from the Human Connectome Project (HCP), freely available in *NeuroVault* (http://neurovault.org/images/3180) [51] (see Additional file 1: Table S1 for MNI coordinates for these ROIs). The following 12 ROIs were identified: medial prefrontal cortex (MPFC), posterior cingulate cortex (PCC), temporoparietal junction (LTPJ, RTPJ), superior temporal sulcus (LSTS, RSTS), inferior frontal gyrus (LIFG, RIFG), angular gyrus (LANG, RANG), and cerebellum (LCEREB, RCEREB). These ROIs were created using spherical binary masks (10-mm radius), and mean parameter estimates averaged across all activated voxels within an ROI were extracted from this same contrast on an individual basis for each ROI to inspect individual variability, and were then statistically compared between the groups using a series of two-sample *t* tests using false discovery rate (FDR) for multiple comparisons correction procedure.

For whole-brain analysis, areas of statistically significant activation differences were determined using one- and two-sample *t* tests using a random-effects model via AFNI’s *3dttest*^*++*^. To correct for multiple comparisons, 10,000 Monte Carlo simulations were applied via AFNI’s *3dClustSim* function to obtain a corrected significance level of *p* < 0.05 (uncorrected voxelwise threshold of *p* < 0.025; minimum cluster size of 100 voxels). In summary, there were two levels of analyses to assess brain activation: ROI and whole-brain analysis.

### Functional connectivity

To examine functional connectivity during each experimental condition, the activation time-series were extracted using AFNI’s *3dmaskave* from the ToM, RD, and GD epochs and were then concatenated to create single time-series for each condition. Several steps of preprocessing attempted to first account for or eliminate several extraneous factors. To minimize signal from the cerebral white matter and lateral ventricles, masks were created at the participant level using FSL’s *FAST* automated segmentation [52]. Masks were trimmed from the white matter to avoid partial-volume effects, and an average time-series for each region was extracted (described below). Derivatives for head motion, white matter, and ventricular time-series were computed. Following spatial smoothing, sources of noise (head motion, white matter, and lateral ventricles plus derivatives) were modeled and removed using a general linear model, and the residual time-series were used in subsequent functional connectivity analysis.

The ROIs mentioned above, derived from *NeuroVault*, were used for functional connectivity analysis. Given the large number of pairwise comparisons across all ROIs, increasing the likelihood of type I error, the ROIs were grouped into five sets based on their anatomical locations: *frontal* (LIFG, RIFG), *medial* (MPFC, PCC), *parietal* (LANG, RANG), *temporal* (LSTS, RSTS, LTPJ, RTPJ), and *cerebellum* (LCEREB, RCEREB). The average time courses were extracted from these sets from individual participants for all three experimental conditions (ToM, GD, and RD) and were correlated across sets to assess the synchronization between them. Correlation coefficients were Fisher’s *z*-transformed, using an inverse hyperbolic tangent function. FDR correction was also applied for all connectivity analyses. In summary, overall functional connectivity and the connectivity among these five functionally defined and anatomically grouped sets of ROIs was measured for each participant separately for each of the ToM, GD, and RD conditions.

#### Accounting for head motion

Because head motion can impact functional connectivity analysis [53, 54], the following precautions were taken. Head motion was quantified as the Euclidean distance calculated from six rigid-body motion parameters (*x*, *y*, *z*, pitch, roll, yaw) for each pair of consecutive time points. For any time point where this measure was >1 mm, which was considered excessive motion, that time point, as well as the immediately preceding and subsequent time points, was modeled out [55]. All participants retained more than 80 % of their time points after censoring, and the number of retained time points did not significantly differ between groups [*M*_TD_ = 663; *M*_ASD_ = 642, *t*(24) = 1.61, *p* = 0.12]. Average head motion over each participant’s session was defined as the root mean square of displacement (RMSD) and did not significantly differ between groups [*M*_TD_ = 0.13; *M*_ASD_ = 0.15, *t*(24) = 1.18, *p* = 0.25].

## Results

### Overview

The main results of this study are the following: (1) significant activation in regions primarily associated with processing ToM and those associated with action understanding (frontal and parietal regions) in both ASD and TD participants; (2) this pattern of activation closely resembles the ToM activation map from the HCP that used a larger sample size; (3) significantly reduced activation on both ROI analysis and whole-brain analysis during ToM processing in the ASD participants, relative to TD peers, in regions that are part of the ToM network and that of action understanding; (4) no significant between-group differences in activation while processing goal-directed animations; and (5) functional underconnectivity in the ASD group relative to the TD group between anatomical networks (fronto-medial, fronto-parietal, and medial-cerebellum).

### Behavioral results

To assess possible group differences in reaction time (RT) and performance accuracy (assessed by error rate) measured during the fMRI task, we conducted two separate two-way (group: TD and ASD) × 3 (condition: ToM, GD, and RD) repeated measures ANOVAs. The first ANOVA revealed a main effect of condition [*F*(2, 48) = 21.4, *p* < .001]; however, there was no significant main effect of group [*F*(1, 24) = 0.6, *p* = 0.5], nor a group × condition interaction [*F*(2, 48) = 0.7, *p* = 0.5], indicating that the groups responded equally in terms of RT.

The second ANOVA testing effects on accuracy of responding revealed a main effect of condition [*F*(2, 48) = 37.6, *p* < .001] and a main effect of group [*F*(1, 24) = 9.5, *p* < .01]; however, the group × condition interaction was not significant [*F*(2, 48) = 1.9, *p* = 0.2]. The fact that the errors in the ASD group were greater than chance in the ToM condition (mean error rate = 81 %) suggests not only that the ASD participants were unable to interpret ToM animations as well as their TD peers (mean error rate = 49 %), but also that they were less likely to select a ToM interpretation than would be expected if they were just guessing. Thus, despite the failure to find a reliable interaction, participants in the ASD group were only outperformed by their TD peers in the ToM condition (see Additional file 1: Table S2) and their bias toward selecting non-ToM descriptions in the ToM condition clearly suggests a deficit in ToM among our sample of children and adolescents with ASD.

### Distribution of fMRI activation

#### Within-group results

**Fig. 1:**
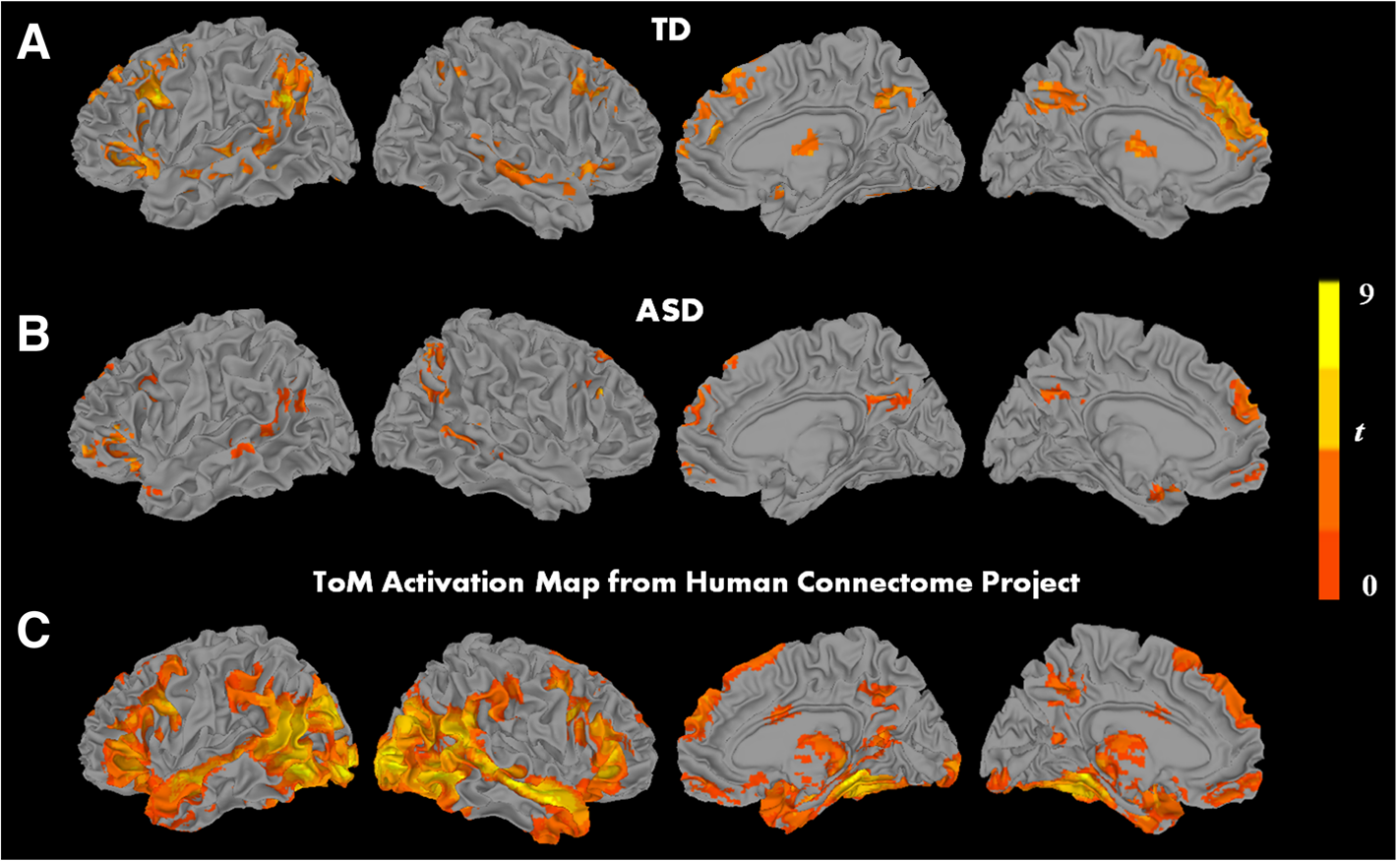
Significant clusters of within-group results for the contrast theory-of-mind vs. random animation (ToM > RD) for the **a** TD group, **b** ASD group, and **c** Human Connectome Project map (*p* < 0.05, FWE corr.)

The processing of ToM animations, when contrasted with RD animations, showed significant activation in both groups in core areas of the ToM network, namely the precuneus/PCC, medial superior frontal gyrus extending to MPFC, and bilateral angular gyrus extending to TPJ, and in additional regions such as bilateral middle temporal gyrus and bilateral IFG and precentral gyrus (see Fig. 1a, b and Additional file 1: Table S3). Processing GD animations, when contrasted with RD animations, showed significant activation in both groups in the middle temporal gyrus and the right IFG (see Additional file 1: Figure S1 and Additional file 1: Table S4). As an additional feature, the HCP ToM activation map, thresholded at the same *t* value as our within-group results, is included in Fig. 1 for comparison (see Fig. 1c). Thus, the ToM task in the current study elicited strong activation in both groups in regions that are part of the ToM network and some regions that are considered part of action understanding.

#### Between-group results

Significant group differences were detected when processing ToM was contrasted with processing RD animations, with the ASD participants showing reduced activation when parameter estimates were compared: MPFC [*t*(24) = 3.30, *p* = 0.003], PCC [*t*(24) = 3.89, *p* = 0.0007], LTPJ [*t*(24) = 3.05, *p* = 0.005], LANG [*t*(24) = 2.27, *p* = 0.04], LIFG [*t*(24) = 2.89, *p* = 0.008], and LCEREB [*t*(24) = 3.46, *p* = 0.002], and no inverse effects (ASD > TD) were found (see Additional file 1: Figure S2).

**Fig. 2:**
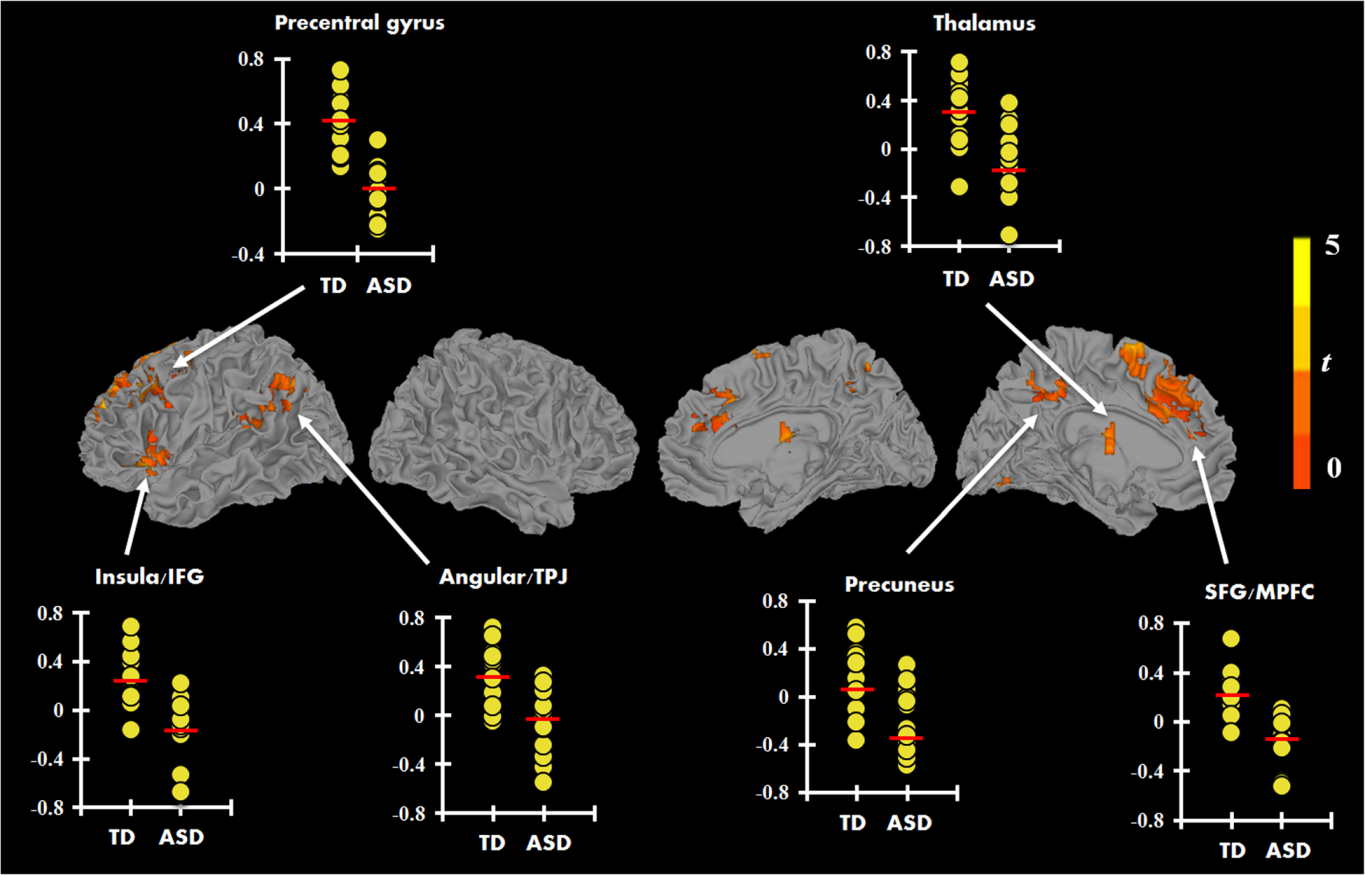
Significant clusters of between-group differences (TD > ASD) for the contrast theory-of-mind vs. random (ToM > RD; *p* < 0.05, FWE corr.), with dot plots showing individual data points for both TD and ASD groups

Furthermore, the whole-brain analysis showed a pattern similar to the ROI analysis, where several left hemisphere areas showed reduced activation in the ASD group compared to the TD such as medial superior frontal gyrus extending to MPFC, precentral gyrus, precuneus/PCC, insula extending to IFG, thalamus, and angular gyrus extending to TPJ (see Fig. 2b, c and Table 2). There was also reduced activity in the cerebellum, specifically in left Crus V1. There was no region where the ASD participants showed greater activation relative to the TD participants in this contrast (i.e., there was no evidence of an ASD > TD effect, for the ToM vs. RD contrast). Additionally, there were no significant group differences in the modulation of activation while processing GD animations compared to the RD condition.

**Table 2:** Group differences in fMRI BOLD activation for ToM vs. random animation and GD vs. random animation (whole-brain analysis)

Contrast and direction	Region	Hemi.	Cluster vol. (in μl)	Peak coordinates MNI			Peak
				*x*	*y*	*z*	*t*
ToM vs. RD	Superior medial gyrus	L	28,593	−4	36	34	5.0
TD > ASD	Insula/inferior frontal	L	7371	−24	22	−12	4.1
	Precentral gyrus	L	6453	−40	4	34	5.0
	Angular gyrus	L	5427	−42	−56	40	4.0
	Cerebellum (Crus VI)	L	3213	−22	−86	−26	4.8
	Precuneus	L	3024	−6	−44	40	3.7
	Thalamus	R	2835	2	−8	18	4.3
ASD > TD	N/A	N/A	N/A	N/A	N/A	N/A	N/A
GD vs. RD							
N/A	N/A	N/A	N/A	N/A	N/A	N/A	N/A

*L* left, *R* right, *Hemi.* hemisphere, *vol.* volume

### Functional connectivity

Functional connectivity analysis revealed a pattern of underconnectivity in the ASD group relative to the TD group in the following way: a two-way (group: TD and ASD) × 3 (condition: ToM, GD, and RD) repeated measures ANOVA was performed to assess overall functional connectivity (by averaging *z*-scores across all ROIs) and revealed no main effects of group [*F*(1, 24) = 0.03, *p* = 0.8] or condition [*F*(2, 48) = 0.65, *p* = 0.53]; however, the group × condition interaction was significant [*F*(2, 48) = 3.43, *p* = 0.04]. Each condition was examined separately, and overall functional connectivity was found to be significantly reduced in the ASD group compared to the TD group only for the ToM condition [*t*(24) = 2.8, *p* = 0.01] but not for GD and RD.

**Fig. 3:**
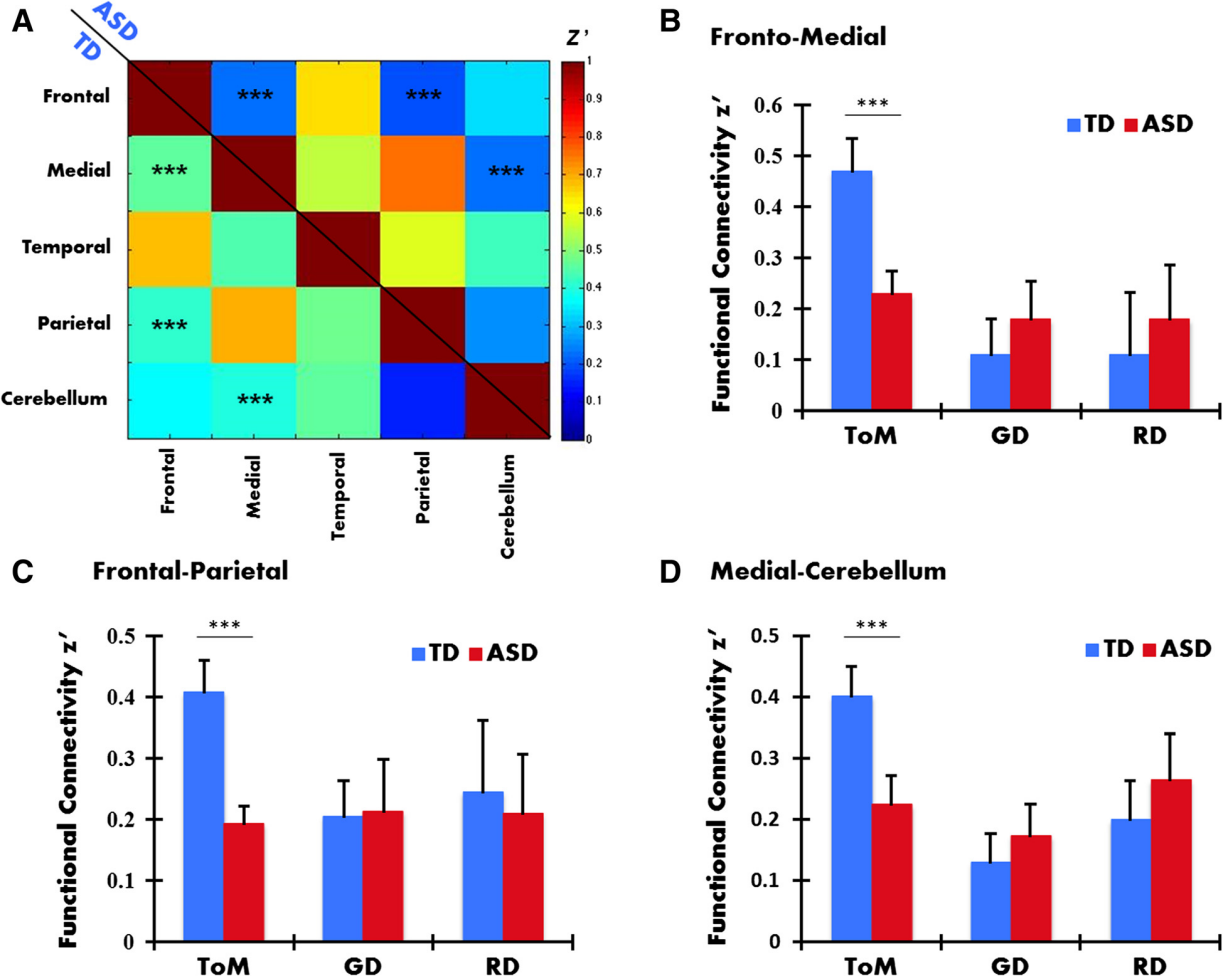
**a** Correlation matrix showing connectivity among frontal, medial, temporal, parietal, and cerebellum networks during ToM condition. Bar graphs for **b** frontal-medial, **c** frontal-parietal, and **d** medial-cerebellum connectivity during ToM, GD, and RD conditions (*error bars* represent SEM; *p < 0.1, uncorrected; **p < .05, uncorrected; ***p < .05, FDR corrected, for the test of the difference in connectivity between groups)

We further examined functional connectivity among anatomical networks (frontal, parietal, temporal, medial, and cerebellum) during the ToM condition (Fig. 3a), revealing significantly decreased connectivity in the ASD group relative to the TD in *frontal-medial* [*t*(24) = 3.90, *p* = .005], *frontal-parietal* [*t*(24) = 3.74, *p* = .001], and *medial-cerebellum* networks [*t*(24) = 2.80, *p* = .01]. It should be noted that this difference was not statistically significant during GD and RD conditions (see Fig. 3b–d), indicating the specificity of our findings of reduced functional connectivity in the ToM condition. We also examined the variability across participants in each group to make sure that the results are not influenced by an outlier or two. This is displayed in Additional file 1: Figure S3.

## Discussion

This study of attributing mental states to animated shapes found robust activation in both ASD and TD groups of participants in regions associated with the ToM network. Additionally, this pattern of activation closely matched the results of an independent and larger dataset from the HCP, where a task similar to ours was used. Analysis of group differences, on the other hand, revealed reduced activation in ASD, relative to TD, participants during the ToM task. This supports the hypothesis of altered/reduced recruitment of the ToM network in ASD, a relatively consistent finding across a range of neuroimaging studies of ToM in autism [10, 20, 21, 23–26]. The activation in the ASD group was reduced in regions associated with processing ToM, including superior frontal gyrus extending to MPFC (SFG/MPFC), angular gyrus extending to TPJ and STS, and precuneus/PCC. Although our cluster of reduced activation in the ASD group in SFG/MPFC was found to be more superior and posterior, and not ventral, it lies very proximate to relatively superior areas in MPFC found in the HCP map. A separate cluster for TD > ASD was found more ventral in MPFC, although this cluster did not survive multiple comparisons correction. These findings of reduced activation in SFG/MPFC, TPJ, STS, and PCC are consistent with neuroimaging studies of ToM using these same animated stimuli in adults with ASD [21, 23]. Additionally, some of the areas of reduced activation in the ASD group for whole-brain analysis overlapped with regions from the HCP map. This was further confirmed as the HCP-based ROI analysis revealed findings similar to our whole-brain results. This underscores the role of these regions in processing ToM, as evidenced from multiple sources of neuroimaging data.

While significant activation in precentral/premotor cortex was observed in the ToM vs. RD contrast (e.g., coaxing) for both groups, this effect was less robust for the GD vs. RD contrast (e.g., chasing), possibly suggesting reliance on motor simulation to *assist* in attribution of mental states to moving objects. Premotor activations have been reported in many previous studies involving action and event prediction [56, 57]. The motor regions may encode the observable, perceptual-motor properties of others (in this case, the triangles dancing or pushing one another), and the ToM system may interpret those properties in terms of unobservable mental states and traits (the big triangle trying to coerce the little one to come out) [6, 58–60]. Additionally, reduced activation was also seen in this same area in the ASD participants, which may suggest limited understanding of the biological and kinematic properties of the animations presented. One possibility that our task elicited additional activation in regions outside of traditional ToM network is that there might be a functional communication among these regions in understanding ToM at richer and more comprehensive levels. This putative functional communication between the ToM network and regions such as the IFG has been observed in studies targeting mentalizing and mirroring abilities [24, 61], and in resting state fcMRI studies in ASD [62], possibly suggesting limited crosstalk among all these regions. It should also be noted that our observed pattern does not conclusively indicate that the ToM network depends on motor simulation for its inputs during high-level ToM processes. It is possible that these two systems may be functionally independent [63]. Further research is needed to directly test for functional interdependence of these systems during higher-level social cognition.

While our activation findings are largely consistent with previous studies, they are inconsistent with the findings from a recent fMRI study of ToM using verbally presented stories requiring drawing inferences with ASD participants in the same age range studied here [27]. These authors reported increased activation in these same regions (MPFC, TPJ, STS, and PCC) and in two different ASD groups (one with high and one with low ToM ability scores) compared to a TD group. They suggested that the age of the participants, the explicitness of the ToM task, or the mental effort required by the task could explain the hyperactivity of the ToM network in ASD they found. Comparison of our results with those of [27] argues against age being the critical factor affecting whether the participants with ASD show over- or under-activation of the ToM network, but we agree that factors related to the ToM task may explain the discrepancies among studies. Although our participants were explicitly instructed to think about what the triangles were doing, the attribution of mental states to the stimuli used here may still be a more implicit mentalizing *process* [64] than that necessary for the verbal reasoning required of the task used by [27]. Recent work has shown that performance in ToM tasks in people with ASD is dependent on whether the measure taps implicit or explicit processing and suggests that explicit verbal reasoning about the thoughts and feelings of others may be an important compensatory strategy in individuals with ASD [65]. For example, tasks that tap more into *explicit* mentalizing processing usually rely more on traditional false-belief tasks [66], where participants are asked to explain the behavior of a story character in a hypothetical scenario. Such processing (explicit) may involve greater recruitment of the ToM network and explain the hyperactivation reported by [27]. We also note that we have previously found increased ToM network activation in adults with ASD when they are required to make inferences about verbally presented stories [25], and that this over-activity in the network occurred regardless of whether the inferences required ToM or not. We suggest that the task used in the present study is sensitive to more implicit processes that are relatively automatic in TD individuals but absent or limited in those with ASD.

Another finding was the reduced activation in the cerebellum, specifically in Crus I, in the ASD group. One of the early accounts that shifted the traditional view of the cerebellum, in sensorimotor processing, was a study by Leiner and colleagues [67] where they presented neuroanatomical, neuroimaging, and behavioral reports of cerebellar involvement in cognitive and language functions. In addition, the cerebellum has been recently proposed to have an important role in social cognition [41]. Examples of tasks that typically trigger activation in this part of the cerebellum involve judgments of intentionality while observing animations of moving shapes [41]. In the current study, these were triangles whose motions evoked interpretations of intentional action/interaction involving thoughts and feelings (e.g., coaxing). The cerebellum has been largely overlooked in social cognition literature in ASD research, despite abnormalities previously reported both structurally and functionally. For example, postmortem studies have revealed abnormalities in Purkinje cells [68–70] and in vivo structural MRI studies have reported hypoplasia of the posterior vermis in ASD [71–73]. Functional MRI studies have found abnormalities in a wide range of tasks in ASD such as language [74], executive functioning paradigms [75], facial and vocal processing [76], and motor performance [77]. More recently, functional connectivity has been explored using both task-based and resting-state targeting low-frequency BOLD signal fluctuations in individuals with ASD [78, 79]. Mostofsky and colleagues found weaker functional connectivity between the cerebellum and motor areas during motor task performance, while Khan and colleagues found weaker functional connectivity between cerebellum and areas associated with higher cognitive functioning (prefrontal, parietal, and temporal) in ASD compared to TD participants. This is similar to the reduced connectivity between medial and cerebellar regions found in our study. The reduced cerebellar activation and connectivity in the ASD participants might underscore the role of the cerebellum in social cognition and suggest a deficit in how the cerebellum is recruited in social processing in ASD.

The finding of reduced functional connectivity in the ASD group is consistent with our previous study with adults using the same experimental paradigm [23] as well as other previous studies [21, 24]. The task-related functional connectivity analysis carried out here revealed significant underconnectivity in children and adolescents with ASD. This is consistent with many studies of people with ASD that have also reported reduced task-induced functional connectivity during processing that included a social or cognitive component (see [28], for an extensive review). As was true of the activation data, group differences in connectivity were observed only for the processing of ToM animations and not during RD or GD animations. This again suggests a fundamental deficit that is unique to ToM in individuals with ASD, and which may result from limited coordination of resources from key regions. Weaker communication among such regions in people with ASD may affect the quality of their ToM abilities, thus affecting the nature and quality of their social interactions [18]. Effective processing of ToM in this task may entail optimal and effective coordination (facilitated by high bandwidth) between relatively distant nodes of the mentalizing network in the TD group. In the ASD group, on the other hand, connectivity may be weaker due to bandwidth constraints between these regions. At the biological level, weaker bandwidth may arise from abnormal white matter trajectories in people with ASD, limiting the degree of synchronization, and this may be more apparent when participants with ASD are asked to perform tasks with high cognitive demand, such as ToM, face perception, language, working memory, and inhibition [28].

One of the limitations of the current study is the relatively smaller sample size, which may have limited our power for detecting stronger effects, in particular, group-by-condition interactions that would lend stronger support to the specificity of the effects to the ToM condition. In addition, the smaller sample size also restricts any analysis of subgroups within the ASD sample. Previous studies have indicated that individuals with higher or lower symptom severity tend to show different connectivity profiles [62, 80]. Nevertheless, the present findings are important, given the dearth of developmental neuroimaging studies in autism [35]. Although there is some evidence of functional over-connectivity in children with ASD [36], most such findings involve low-frequency fluctuations as opposed to an active cognitive task in the current study. In addition, most studies reporting underconnectivity in ASD have addressed domains such as cognitive (working memory, problem-solving, response inhibition), social (ToM, biological motion, face processing), and language (discourse processing, prosody, pun, irony, sentence comprehension, semantic processing), where individuals with ASD tend to show some degree of impairment. Methodological choices have also played a role in the disparity of findings while comparing children vs. adults [37]. Therefore, longitudinal studies tracking the same individuals as they progress from pre-to post-pubertal stages of development are needed to gain a better understanding of the neurodevelopmental trajectory in autism.

## Conclusions

In summary, our results showed reduced activation in core ToM regions and non-traditional ToM regions, and reliable underconnectivity across several networks thought to be involved in ToM. The findings of this study provide valuable insights into the neurobiology of social cognition in autism, especially to the complex profile of brain activation and connectivity in children with autism. They also shed light on the disruption in brain functioning in general and connectivity in particular in autism when challenged by complex tasks like ToM.

## Additional file

Additional file 1: Table S1. Seed ROI coordinates for fcMRI analysis. **Table S2.** Reaction time and error rate for all experimental conditions. **Table S3.** fMRI BOLD activation for ToM vs. random animation. Table S4. fMRI BOLD activation for GD vs. random animation. **Figure S1.** Within-group results for the contrast goal-directed vs. random animation for the a) TD group, and b) ASD group (*p* < 0.05, FWE corr.). **Figure S2.** Mean dot plots of each ROI for both TD and ASD groups for the contrast theory-of-mind vs. random (**p* < 0.1, uncorrected; ***p* < .05, uncorrected; ****p* < .05, FDR corrected, for the test of the difference in activation between groups). **Figure S3.** Mean dot plots for B) frontal-medial, C) frontal-parietal, and D) medial-cerebellum connectivity during ToM, GD, and RD conditions (**p* < 0.1, uncorrected; ***p* < .05, uncorrected; ****p* < .05, FDR corrected, for the test of the difference in connectivity between groups).

## Abbreviations

ADI: Autism Diagnostic Interview
ADOS: Autism Diagnostic Observation Schedule
ANOVA: analysis of variance
ASD: autism spectrum disorder
BOLD: blood-oxygen-level dependent
fcMRI: functional connectivity MRI
FDR: false discovery rate
FMRI: functional magnetic resonance imaging
FWHM: full width at half maximum
GD: goal-directed
GDQ: goal-directed question
GLM: general linear model
HCP: human connectome project
IQ: intelligence quotient
LANG: left angular gyrus
LCEREB: left cerebellum
LIFG: left inferior frontal gyrus
LSTS: left superior temporal sulcus
LTPJ: left temporoparietal junction
MNI: Montreal Neurological Institute
MPFC: medial prefrontal cortex
PCC: posterior cingulate cortex
RANG: right angular gyrus
RCEREB: right cerebellum
RD: random
RDQ: random question
RIFG: right inferior frontal gyrus
RMSD: root mean square of displacement
ROI: region of interest
RSTS: right superior temporal sulcus
RT: reaction time
RTPJ: right temporoparietal junction
TD: typically developing
ToM: theory-of-mind
ToMQ: ToM question
