# Supplementary material for: Aberrant functioning of the theory-of-mind network in children and adolescents with autism
Source: Mol Autism. 2015 Oct 27;6:59. doi: 10.1186/s13229-015-0052-x (PMC4624365; doi:10.1186/s13229-015-0052-x)

**Supplementary Tables**

| **Supplementary Table 1. Seed coordinates for fcMRI analysis** | | | |
| --- | --- | --- | --- |
|  | **Peak coordinates MNI** | | |
| **Seed** | **x** | **y** | **z** |
|  |  |  |  |
| Medial prefrontal Cortex (MPFC) | 0 | 48 | -18 |
| Posterior cingulate cortex (PCC) | 0 | -52 | 22 |
| Left superior temporal sulcus (LSTS) | -58 | -48 | 7 |
| Right superior temporal sulcus (RSTS) | 58 | -44 | 8 |
| Left temporoparietal junction (LTPJ) | -58 | -58 | 22 |
| Right temporoparietal junction (RTPJ) | 58 | -52 | 20 |
| Left inferior frontal gyrus (LIFG) | -48 | 14 | 26 |
| Right inferior frontal gyrus (RIFG) | 48 | 14 | 26 |
| Left angular gyrus (LANG) | -46 | -72 | 31 |
| Right angular gyrus (RANG) | 48 | -69 | 31 |
| Left Cerebellum (LCEREB) | -20 | -72 | -36 |
| Right Cerebellum (RCEREB) | 20 | -72 | -36 |

| **Supplementary Table 2. Reaction time and error rate for all experimental conditions** | | | | | | | |
| --- | --- | --- | --- | --- | --- | --- | --- |
|  | **Reaction time (ms)** | | |  | **Mean error rate (%)** | | |
| **Group** | **RD** | **GD** | **ToM** |  | **RD** | **GD** | **ToM** |
|  |  |  |  |  |  |  |  |
| TD | 5699 | 4555 | 5887 |  | 18 | 5 | 49 |
|  | (±1625) | (±1444) | (±1434) |  | (±17) | (±13) | (±32) |
|  |  |  |  |  |  |  |  |
| ASD | 5343 | 3842 | 5816 |  | 28 | 14 | 81 |
|  | (±1573) | (±1206) | (±1669) |  | (±30) | (±23) | (±23) |
| Values are presented as mean (standard deviation). | | | | | | | |

| **Supplementary Table 3. fMRI BOLD activation for ToM vs. Random Animation** | | | | | | | |
| --- | --- | --- | --- | --- | --- | --- | --- |
|  |  |  | **Cluster vol.** | **Peak coordinates MNI** | | | **Peak** |
| **Group** | **Region** | **Hemi.** | **(in µl)** | **x** | **y** | **z** | ***t*** |
| **TD** | Medial superior gyrus | L | 46062 | -7 | 49 | 19 | 7.3 |
|  | Angular gyrus | L | 29862 | -52 | -57 | 28 | 9.8 |
|  | Precentral gyrus | L | 26514 | -40 | 4 | 37 | 8.3 |
|  | Insula | R | 19413 | 42 | 22 | -9 | 5.6 |
|  | Cerebellum | R | 15336 | 30 | -81 | -27 | 7.3 |
|  | Precuneus | R | 11556 | 3 | -63 | 43 | 5.4 |
|  | Middle frontal gyrus | R | 9936 | 42 | 25 | 34 | 5.9 |
|  | Caudate nucleus | L | 8262 | -13 | 7 | 19 | 6.5 |
|  | Angular gyrus | R | 4590 | 42 | -57 | 37 | 6 |
| **ASD** | Medial superior gyrus | L | 7749 | -1 | 52 | 28 | 4.3 |
|  | Inferior frontal gyrus | L | 5508 | -43 | 37 | -6 | 4 |
|  | Middle temporal gyrus | L | 5400 | -46 | -51 | 22 | 3 |
|  | Middle occipital gyrus | R | 4752 | 57 | -66 | 25 | 4.7 |
|  | Cuneus | L | 2700 | -1 | -69 | 31 | 2.9 |
|  | Precentral gyrus | L | 1701 | -46 | 13 | 34 | 2.6 |
|  | Superior frontal gyrus | R | 1701 | 18 | 34 | 52 | 3.4 |
|  | Inferior frontal gyrus | R | 1620 | 45 | 16 | 31 | 2.6 |
|  | Middle temporal gyrus | R | 1593 | 66 | -51 | 4 | 2.7 |
|  | Mid orbital gyrus | L | 1512 | -1 | 61 | -9 | 2.7 |
|  | Amygdala | L | 1377 | -25 | 4 | -24 | 3.6 |

| **Supplementary Table 4. fMRI BOLD activation for GD vs. Random Animation** | | | | | | | |
| --- | --- | --- | --- | --- | --- | --- | --- |
|  |  |  | **Cluster vol.** | **Peak coordinates MNI** | | | **Peak** |
| **Group** | **Region** | **Hemi.** | **(in µl)** | **x** | **y** | **z** | ***t*** |
| **TD** | Middle temporal gyrus | R | 9531 | 66 | -45 | 10 | 5.2 |
|  | Inferior occipital gyrus | L | 7317 | -43 | -75 | -9 | 6.1 |
|  | Calcarine gyrus | R | 6372 | 24 | -93 | 1 | 5.4 |
|  | Cerebellum | L | 2484 | -19 | -72 | -30 | 4.9 |
|  | Inferior frontal gyrus | R | 2214 | 54 | 25 | 13 | 7.1 |
|  | Middle temporal gyrus | L | 2025 | -61 | -60 | 19 | 5 |
|  | Thalamus | R | 1863 | 12 | -30 | -3 | 3.7 |
| **ASD** | Fusiform gyrus | L | 24840 | -43 | -60 | -15 | 8.4 |
|  | Inferior temporal gyrus | R | 23220 | 48 | -60 | -6 | 6 |
|  | Angular gyrus | R | 11475 | 33 | -66 | 46 | 6.6 |
|  | Supramarginal gyrus | R | 9072 | 57 | -30 | 25 | 5.7 |
|  | Lingual gyrus | R | 7884 | 6 | -33 | -6 | 5.1 |
|  | Hippocampus | R | 4914 | 27 | -15 | -12 | 5.7 |
|  | Inferior frontal gyrus | R | 3591 | 45 | 13 | 25 | 5.9 |
|  | Middle temporal gyrus | L | 2133 | -61 | -24 | 1 | 4.4 |
|  | Middle frontal gyrus | R | 1863 | 45 | 46 | 19 | 3.9 |
|  | Amygdala | L | 1620 | -28 | 4 | -18 | 4.4 |
|  | Inferior frontal gyrus | L | 1539 | -52 | 34 | 13 | 5.6 |

**Supplementary Figures**

**Supplementary Figure 1.** Within-group results for the contrast Goal-Directed vs. Random Animation for the a) TD group, and b) ASD group (*p* < 0.05, FWE corr.).

**
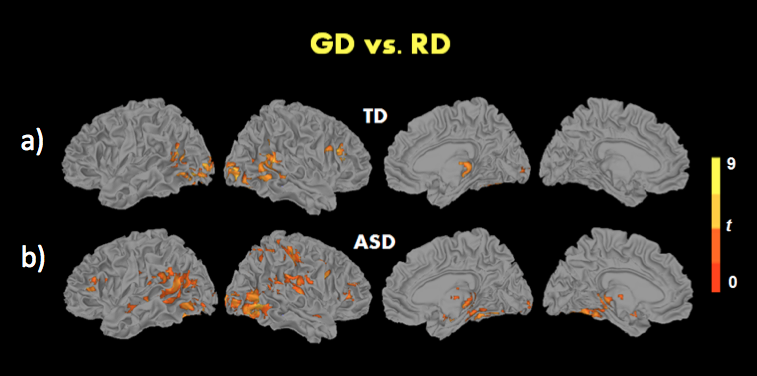
**

**Supplementary Figure 2.-** Mean-dot plots of each ROI for both TD and ASD groups for the contrast Theory-of-Mind vs. Random (**p* < 0.1, uncorrected; ***p* < .05, uncorrected; ****p* < .05, FDR corrected, for the test of the difference in activation between groups).


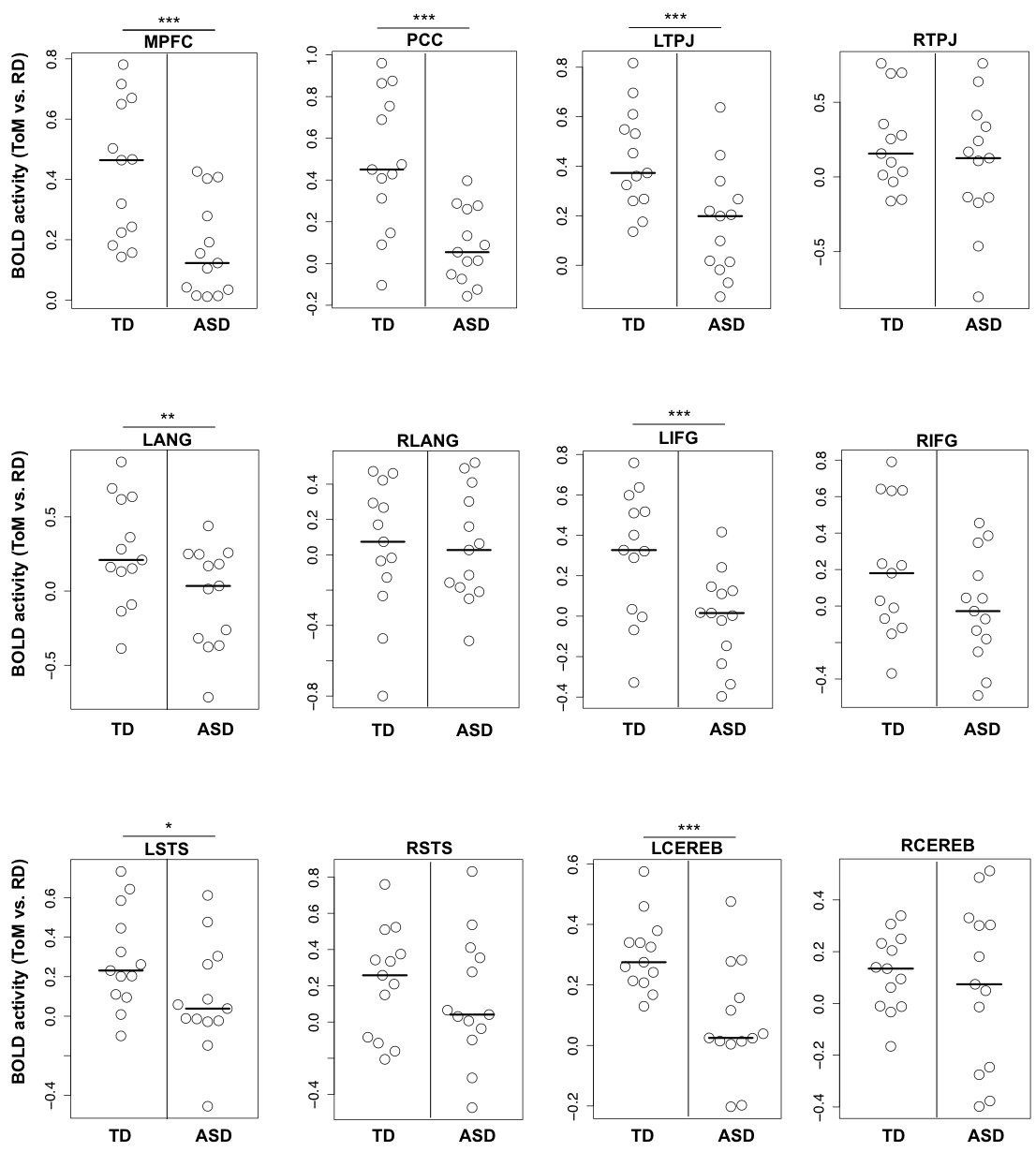


**Supplementary Figure 3.-** Mean-dot plots for B) Frontal-Medial, C) Frontal-Parietal, and D) Medial-Cerebellum connectivity during ToM, GD, and RD conditions (**p* < 0.1, uncorrected; ***p* < .05, uncorrected; ****p* < .05, FDR corrected, for the test of the difference in connectivity between groups).
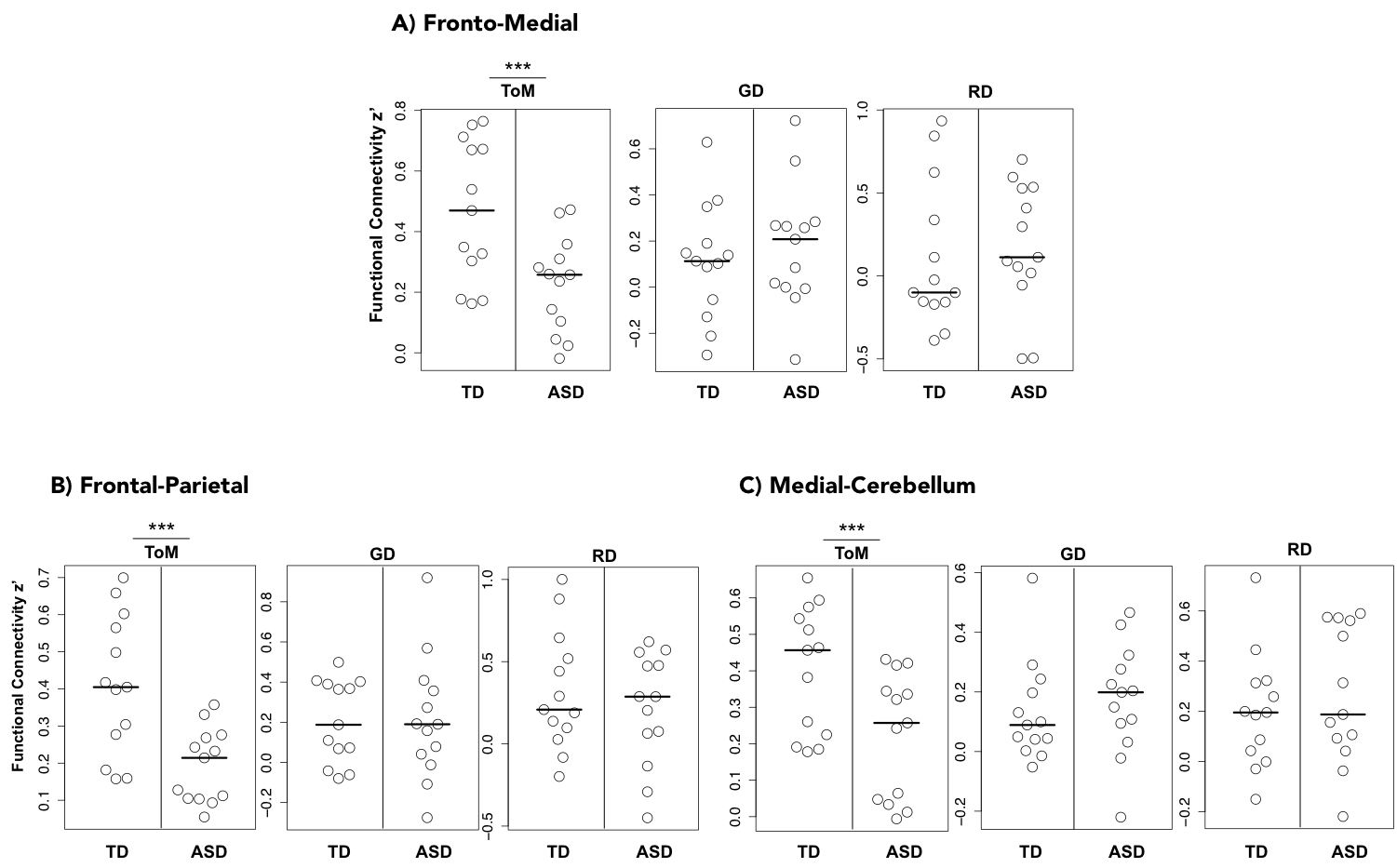

Supplement: Additional file 1: Table S1. — Seed ROI coordinates for fcMRI analysis. Table S2. Reaction time and error rate for all experimental conditions. Table S3. fMRI BOLD activation for ToM vs. random animation. Table S4. fMRI BOLD activation for GD vs. random animation. Figure S1. Within-group results for the contrast goal-directed vs. random animation for the a) TD group, and b) ASD group (p < 0.05, FWE corr.). Figure S2. Mean dot plots of each ROI for both TD and ASD groups for the contrast theory-of-mind vs. random (*p < 0.1, uncorrected; **p < .05, uncorrected; ***p < .05, FDR corrected, for the test of the difference in activation between groups). Figure S3. Mean dot plots for B) frontal-medial, C) frontal-parietal, and D) medial-cerebellum connectivity during ToM, GD, and RD conditions (*p < 0.1, uncorrected; **p < .05, uncorrected; ***p < .05, FDR corrected, for the test of the difference in connectivity between groups). [file 13229_2015_52_MOESM1_ESM.docx]
